# Costs and Effects of School-Based Licensed Practical Nurses on Elementary Student Attendance and Chronic Absenteeism

**DOI:** 10.1007/s11121-022-01459-0

**Published:** 2022-11-17

**Authors:** Stephen M. Leach, Fiona M. Hollands, Eva Stone, Robert Shand, Laura Head, Yixin Wang, Bo Yan, Dena Dossett, Florence Chang, Yuan Chang Ginsberg, Yilin Pan

**Affiliations:** 1 Jefferson County Public Schools, Louisville, KY 40218 USA; 2 Teachers College, Columbia University, New York, NY 10027 USA; 3 American University, Washington, DC 20016 USA; 4 UNICEF, New York, NY USA; 5 World Bank, Washington, DC 20433 USA

**Keywords:** School nursing, Cost-effectiveness analysis, Attendance, Chronic absenteeism, Licensed practical nurse

## Abstract

Despite increasing reliance on licensed practical nurses (LPNs) to provide health services in schools, we do not know whether this is a cost-effective prevention strategy against student absenteeism. Therefore, we evaluated the costs and effectiveness of an LPN-based school nursing program for improving attendance and chronic absenteeism at a large, urban school district in the southeastern USA. We first identified a matched set of 46 elementary schools (23 nurse, 23 no-nurse) by using an optimal multilevel matching algorithm based on student- and school-level characteristics. We then conducted a cost-effectiveness analysis on the matched set, using the ingredients method to estimate societal costs and multilevel regression to estimate effects. The results indicated that despite substantial incremental costs of $68,228 per school, the presence of a full-time LPN was associated with at best negligible improvements, and at worst slight disimprovements, in attendance and chronic absenteeism. We recommend a careful review of the theory of change for LPN-based school nursing programs to clarify the specific inputs and activities that are expected to lead to improved student outcomes. Education agencies should develop explicit assignment, training, monitoring, and auditing plans to ensure LPNs are equitably distributed and that their activities are aligned with the theory of change. Education agencies should also explore whether expanded Medicaid billing can reduce their share of the nursing cost burden.

**Supplementary Information:**

The online version contains supplementary material available at 10.1007/s11121-022-01459-0.

## Introduction

Absenteeism is widely recognized as an important deterrent to student success (Darnell et al., 2019; London et al., 2016). For elementary students in particular, reduced attendance (i.e., higher absenteeism) has been linked to lower grades (Koçoğlu & Emiroglu, 2017) and lower standardized test scores (Gottfried, 2013). The negative effects on achievement are even stronger when elementary students are chronically absent (Gottfried, 2014). Furthermore, chronically absent students—that is, those missing at least 10% of instructional days—are more likely to be chronically absent in subsequent school years (London et al., 2016). Thus, it is not surprising that the American Academy of Pediatrics (2019) has strongly emphasized the prevention of chronic absenteeism as a key strategy for improving students’ academic achievement and long-term health and employment outcomes.

Evidence from descriptive studies suggests that school nurses can play a vital role in improving attendance (e.g., Best et al., 2018; Darnell et al., 2019; Yoder, 2020) and reducing chronic absenteeism (e.g., Koçoğlu & Emiroglu, 2017). School nurses interviewed by Rankine et al. (2021) identified key activities for addressing chronic absenteeism along all four domains of the National Association of School Nurses’ (NASN, 2016) best practices framework: *care coordination* (e.g., case management); *leadership* (e.g., helping create attendance policy); *quality improvement* (e.g., data-based attendance interventions); and *community and public health* (e.g., health education). Notably, those activities are the focus of baccalaureate nursing education and are core competencies for registered nurses (RNs); hence, the NASN (2021) recommends that every school have a baccalaureate-trained RN. However, only about 84% of public schools employed a nurse in 2020 (Buttner, 2021), and though most school nursing programs consist solely of RNs, reliance on licensed practical nurses (LPNs) is more common in the South (Willgerodt et al., 2018).

Best et al. (2018) used the NASN framework to review 65 mostly descriptive (80%) studies of school nursing interventions. They concluded the preponderance was related to care coordination, although only about one quarter of those studies investigated student outcomes related to school nursing. Studies that did include student outcomes were limited by relatively weak study designs. For example, Engelke et al. (2014) and Carpenter et al. (2013) found that nurses reduced parent-reported tardiness and absences related to asthma, but Engelke et al. did not control for confounding factors and neither study included a control group. In perhaps the most rigorous study to date, Gottfried (2013) found nurse presence was associated with decreased absences among urban elementary school students after controlling for students’ gender, race, English learner status, special education status, and reading and math achievement. In general, however, research linking school nurse presence to attendance outcomes lacks sufficient rigor (Yoder, 2020). Both Yoder and Best et al. called for studies with more rigorous correlational and experimental research designs to further our understanding of the relationship between school nursing and student academic outcomes. Best et al. also encouraged using academic outcome variables with standardized definitions (e.g., absenteeism) and dissemination of nonpositive findings to improve school nursing practice and strengthen future research.

Despite the growing body of evidence supporting school nursing as a potentially effective means of preventing absenteeism, gaps remain in our understanding of school nursing as a strategy for addressing this issue. In general, the costs of school nursing programs are not well-described in the literature. Baisch et al. (2011) estimated that a large, urban school district in the Midwest saved over $60,000 per school in 2006–2007 by using nurses instead of school staff to provide health services. However, their analysis did not include costs of other nursing-related administrative personnel or costs of medical supplies and facilities. Wang et al. (2014) conducted a cost–benefit analysis of services provided by baccalaureate-trained RNs. Using Massachusetts prices, they estimated the cost per school of providing a full-time RN to be $84,724 in 2009 dollars. Although Wang et al. took a more comprehensive approach by computing costs from a societal perspective, they did not document all resources used to implement the nurse program and their comparison was based on a hypothetical no-nurse condition. Neither study specifically addressed school nurse effects on absenteeism. Despite the call by Bensink et al. (2013) for cost-effectiveness analyses (CEAs) in nursing studies, researchers to date have not conducted a detailed CEA (e.g., Levin et al., 2018) of a school nurse program using actual cost and effectiveness data for both treatment and comparison conditions.

Given the prevalence of RN-only school nurse programs (Willgerodt et al., 2018) and the NASN’s (2021) school nurse recommendations, the focus on RNs in previous studies (e.g., Allen, 2003; Jacobsen et al., 2016; Rankine et al., 2021) is not surprising. Despite the NASN’s urging, however, only about 40% of schools employ a full-time RN (Buttner, 2021), and nearly one-fifth of all school nurse programs in the USA—and more than-one quarter in the South—are staffed partially or entirely by LPNs (Willgerodt et al.). Ongoing nursing shortages, exacerbated by the challenges of responding to COVID-19, have led more schools to hire LPNs since 2019 (Buttner, 2021). Given the differences between LPNs and RNs in both training and scope of duties, especially leadership and care coordination (e.g., NASN, 2021), we do not know whether previously discovered positive effects of RNs on elementary student attendance (e.g., Allen, 2003; *d* = 0.3) and chronic absenteeism (e.g., Jacobsen et al., 2016; 5–8% reductions schoolwide) also apply to LPNs and justify investing substantial resources in school-based LPN programs.

Thus, we evaluated the costs and effects of an existing LPN-based school nurse program during the 2018–2019 school year in a large, urban district in the southeastern USA. We assessed impact on attendance and chronic absenteeism, hypothesizing that full-time LPN presence would be associated with higher costs, higher attendance, and lower chronic absenteeism, compared with schools providing basic health services delivered by unlicensed assistive personnel (UAP). Given the limited scope of LPN training and services offered compared with RNs (e.g., Buttner, 2021; Winsch, 2016), we expected weaker effects on attendance and chronic absenteeism than observed in previous studies investigating full-time, RN-based school nurse programs.

## Method

The Cost Analysis Standards Project (CASP) (2021) recommends estimating incremental costs and effects through separate but related analyses of a program under examination in a CEA. Thus, after presenting information applicable to both analyses, we separately outline our methods for estimating the effects of the LPN-based program and our methods for estimating its costs.

### Design

We studied an existing condition in which all schools provided basic health services while only a few employed a nurse. Our retrospective study used data collected as part of normal routines, necessitating a quasi-experimental design because neither assignment of school nurses to select schools nor student enrollments were randomized. To approximate an RCT more closely and because our data were nested (i.e., students in schools), we used optimal multilevel matching (OMM; Pimentel et al., 2018) to produce a set of highly comparable treatment and comparison schools. Treatment schools were served by a full-time LPN while comparison schools provided only the required basic health-related services administered by UAPs.

### Setting and Participants

Jefferson County Public Schools (JCPS) is a large, urban school district in Louisville, KY. With 6,188 teachers serving 98,359 students in 167 school sites and an annual budget of $1.7 billion in 2018–2019, JCPS is by far the largest district in Kentucky and one of the largest in the nation. That year, the district’s students were 43% White, 36% Black, 12% Hispanic/Latinx, and 9% other races. Nearly two-thirds of JCPS schools were eligible for Title I funding in 2018–2019, and roughly 65% of students were eligible for free or reduced-price lunch (FRL).

All JCPS schools are required, at a minimum, to provide basic health services via UAPs. In 2018–2019, 35 JCPS schools were staffed by a full-time nurse, mostly LPNs (*n* = 30), while 132 schools had no full-time nurse. Six Advanced Practice Registered Nurses (APRNs) supervised the health service provisions by nurses and UAPs in their respective APRN zones. Although most JCPS schools with a full-time nurse were high-needs (e.g., Title I-eligible), the district did not have a systematic process in place for assigning nurses. At the time of the study, student health information was entered into the district’s existing web-based student information system rather than an electronic health record, limiting JCPS’ ability to capture detailed student health data.

### Inclusion and Exclusion

Among the 30 schools staffed by a full-time LPN in the 2018–2019 school year, 25 were elementary schools, three were high schools, and two were K-12 schools for students with special needs. We did not include the two K-12 schools in our study because we would not be able to find matched comparison schools. We also excluded the high schools because the options for finding matched comparison schools were limited by the smaller number of high schools overall. From the remaining 67 elementary schools, we excluded seven as potential matches; one school had a full-time RN, one school was taking part in a pilot RN program, and five schools had part-time nurses. This left a pool of 60 schools with no LPN as potential matches for our 25 elementary schools with a full-time LPN. Our effectiveness analyses excluded kindergarten students due to lack of prior-year pretest data and any students with total enrollment less than 10 days (based on standard district procedures). Our cost analysis included the costs of providing school-based health services to all students enrolled in each school in the final matched set.

### Sampling Procedures

To account for overt biases in multilevel observational studies, Pimentel et al. (2018) developed an OMM algorithm that permits dynamic balancing at the cluster and individual level. The algorithm is said to be *optimal* in that it minimizes the set of distances between level 2 clustered units and level 1 individual units and *dynamic* in that it permits the exclusion of level 2 clustered units to obtain the largest possible balanced matched set (Pimentel et al., 2018). Adequate balance is obtained when matched absolute standardized differences (*asd*s) on level 2 covariates of interest are < 0.20 (e.g., Rosenbaum, 2020).

To obtain a matched set of schools, we used the R package *matchMulti* (Pimentel et al., 2018) to first match schools (not students) on five student-level covariates: binary indicators of minority, FRL, special education, and English learner statuses, and prior-year attendance rate. We then fine balanced the set using six partitioned (*n* = 2, *tolerance* = 0.20) school-level demographic (e.g., Baisch et al., 2011) and pretest covariates: need index (a weighted index of the percent of a school’s students who received special education services, were FRL-eligible, English learners, and mobile—that is, enrolled in two or more schools during the year), mean prior-year attendance, percent of students classified as minority, percent of students chronically absent in the prior year, percent of students rated as novices (i.e., low proficiency) in reading on the prior year state assessment, and percent of students rated as novices in math on the prior year state assessment. Based on consultation with JCPS’s Chief of Accountability, Research, and Systems Improvement and the Manager of District Health, priority was given to obtaining balance on the first three variables. This fine balance approach produced the largest matched set with acceptable balance (*asd* = 0.13 to 0.19) between treatment (*n* = 23) and comparison (*n* = 23) schools on the three prioritized school-level covariates. We excluded two elementary schools with a full-time LPN for which we could not identify a suitable match.

The 23 schools in our sample with a full-time LPN served fewer students on average (*M* = 449.48) than the 23 schools without a nurse (*M* = 478.39). Including enrollment in the OMM algorithm did not increase the size of the matched set and resulted in worse balance on other covariates; thus, we controlled for enrollment in our regression models. Treatment schools were slightly more likely (22 of 23 vs. 21 of 23) to receive Title I funding than comparison schools and had a marginally higher percentage of students who were FRL-eligible (80% vs. 78%). Treatment school students (*n* = 10,338) and comparison school students (*n* = 11,003) were 45% Black, 30% White, 16% Hispanic/Latinx, and 10% other races. Roughly 20% of students in schools with a full-time LPN had at least one recorded health condition versus 14% of students in non-nurse schools. Including health conditions as a matching covariate would reduce the matched set to 30 schools, so instead we controlled for health conditions in subsequent analyses.

### Estimating the Effects of School Nursing

#### Measures and Covariates

##### Outcome Variables

We estimated two separate models to investigate the effects of LPN presence on two student-level outcomes: *attendance* (continuous) and *chronic absenteeism* (dichotomous)*.* Attendance was measured as a student’s days attended divided by their total days enrolled, with possible values ranging from 0 to 1. We defined chronic absenteeism (0 = no, 1 = yes) as a student missing 10% or more instructional days (i.e., attendance ≤ 90%).

##### Covariates

Student-level covariates for each model were *prior year* (i.e., 2017–2018) *attendance* or *chronic absenteeism*; *minority status* (0 = no, 1 = yes); *gender* (categorized as 0 = female, 1 = male); *FRL status* (0 = paid, 1 = free/reduced); *special education status* (0 = no, 1 = yes); *English learner (EL) status* (0 = no, 1 = yes); and an indicator of a student having at least one recorded chronic *health condition* (0 = no, 1 = yes). School-level covariates were *enrollment*, *need index*, *percent minority*, *prior year attendance*, *prior year chronic absenteeism*, *percent reading novice*, *percent math novice*, and *nurse*—a binary indicator of having a full-time LPN (0 = no, 1 = yes). *Percent reading novice* (*asd* = 0.44) and *percent math novice* (*asd* = 0.38) were the only two school-level matching variables inadequately balanced after matching.

#### Analytic Approach

To evaluate the effects of a full-time LPN on attendance and chronic absenteeism, we estimated separate multilevel regression models for each outcome for our matched set of schools using the R package *lme4* (Bates et al., 2015). Page et al. (2020) recommend an OMM + multilevel regression approach to assessing school-wide intent-to-treat effects in clustered observational studies. Although our data were clustered, we assessed the appropriateness of multilevel regression by calculating design effects (DEs; e.g., Stormshak et al., 2021) for attendance and chronic absenteeism. As per Lai and Kwok (2015), we interpreted DE > 1.5 to support a multilevel modeling approach because we were interested in the (level 2) effect of school nurses on (level 1) student attendance and chronic absenteeism.

For each outcome, we first estimated a random intercepts model with student-level covariates (*prior year attendance* or *chronic absenteeism*, *minority status*, *gender*, *FRL status*, *special education status, EL status*, and *health conditions*). Next, we estimated models adding school-level covariates (*enrollment*, *need index*, *percent minority*, *prior year attendance*, *prior year chronic absenteeism*, *percent reading novice*, *percent math novice*, and *nurse*). We then compared fit with modified versions of our respective preferred models that allowed *prior year attendance* or *chronic absenteeism* slopes to vary randomly.

We used the *lmer* command (*REML* = False) for our models with the continuous attendance outcome and *glmer* (family = binomial(logit)) for our binary chronic absenteeism outcome. Based on convention (e.g., Mulawa et al., 2018), we group mean centered student-level continuous variables and grand mean centered school-level continuous variables. Because enrollment values were on a drastically different scale, we standardized values using the *scale* command. Statistical significance was determined by *p* values < 0.05; however, we also report confidence intervals obtained via the *confint* command for our primary coefficients of interest.

### Estimating the Costs of School Nursing

We used the ingredients method (Levin et al., 2018) to estimate the societal costs of providing nursing and health-related services to students in the 46 schools participating in the study. This approach required the identification of all personnel, materials, equipment, and facilities utilized by the JCPS district office, schools, families, and volunteers in the implementation of the school health program and an accounting of their opportunity costs. We determined the type, quantity, and price of each ingredient used to provide nursing and health-related services. As recommended by Hollands et al. (2021) and CASP (2021), we collected data on resource use from all 46 sites. In addition, we identified both local prices to inform district decision-makers and national average prices to provide results that would be applicable to a wider audience and be directly comparable to other cost studies of educational programs. All prices were inflation-adjusted to 2018 dollars for consistency and alignment with the effectiveness results from the same year. We estimated average costs per school of providing LPNs in the treatment schools and of providing only basic health-related services administered by UAPs in the comparison schools. We also estimated costs at each individual school in order to provide a range. To obtain the incremental costs of providing LPNs compared with only basic health services, we subtracted the comparison school costs from the treatment school costs. These incremental costs align with the effects estimated for the same set of schools.

To determine the types and quantities of ingredients used to provide health services at JCPS, we initially reviewed an existing evaluation report on the JCPS school nurse program (Winsch, 2016). We subsequently conducted multiple interviews with the Manager of District Health and exchanged many emails with her to gather information about personnel time use, facilities used for training and service delivery, and school health supplies and equipment. We obtained training rosters listing the individuals from each school who received training to serve as UAPs and the number of hours for each person, invoices for contract nurse services at each school, and inventories of health supplies and equipment from schools and the JCPS warehouse.

Personnel time for district and school employees for our local cost estimate was valued using the JCPS schedule of salaries and hours of work per year by position type, a public database of JCPS personnel annual salaries in 2018, and the district’s 2018 fringe rate calculator. For our national average cost estimate, we obtained national salaries and fringe benefits rates for the relevant positions from the Bureau of Labor Statistics. When national prices for equivalent ingredients were unavailable, we adjusted the local price to a national equivalent using a regional price parity from the Bureau of Economic Analysis. Prices for materials and equipment were sourced from online suppliers or the JCPS warehouse, and we assumed that local and national prices were the same. Items that could be used for multiple years were amortized over 2–10 years. Opportunity costs of facilities were estimated using construction prices of elementary schools and medical office buildings amortized over 30 years using US Treasury bond yields as an interest rate. Construction prices were obtained from a national project management company. The closest local construction prices were for Raleigh Durham, NC. For national construction prices, we averaged prices from all 20 locations across the USA provided by this source.

To conduct the cost analysis, we used the CAP Project’s Excel-based cost analysis template, CAPCAT 1.3 (Hollands et al., 2022). The resulting workbook, provided as a supplement (available online), shows line-by-line ingredients lists indicating the quantity of each resource needed (see Personnel, Materials, and Facilities tabs); the price used to value the resource and source of this price; price adjustments; and local and national costs. We conducted a sensitivity analysis to assess the extent to which our results changed when we increased the amount of time UAPs spent providing basic health services in the comparison schools. In our base case analysis, we estimated this at 4 h per week for each of two UAPs, based on information from our interview with the Manager of District Health. In the sensitivity analysis, we increased this to 6.5 h per week per UAP based on Baisch et al. (2011) finding that various school staff in schools without a nurse collectively spent 13 h per week on health issues.

## Results

### Effectiveness

Table 1 provides descriptive statistics for the final effectiveness sample (*n* = 16,025). The sample did not include kindergartners, students enrolled for fewer than 10 days (*n* = 60), or 610 students (266 nurse, 344 no-nurse) who were missing pretest data due to non-enrollment in JCPS in 2017–2018. The average cluster size was 348.37. Design effects for attendance (*ICC* = 0.0167; *DE* = 6.80) and chronic absenteeism (*ICC* = 0.0198; DE = 7.88) suggested multilevel analyses were necessary to obtain unbiased school-level regression coefficients (e.g., Lai & Kwok, 2015).

**Table 1:** Descriptive statistics for effectiveness sample (*n* = 46 schools; 16,025 students in grades 1–5)

Variable	LPN schools (*n* = 23 schools) (*n* = 7,932 students)	No-nurse schools (*n* = 23 schools) (*n* = 8,093 students)
**Student-level**		
17–18 attendance (M/SD)	0.94 (0.05)	0.94 (0.05)
18–19 attendance (M/SD)	0.94 (0.05)	0.95 (0.05)
17–18 chronic absenteeism (#/% students)	1,168 (15%)	1,194 (15%)
18–19 chronic absenteeism (#/% students)	1,148 (14%)	1,068 (13%)
Black (#/% students)	3,478 (44%)	3,790 (47%)
Hispanic/Latinx (#/% students)	1,314 (17%)	1,137 (14%)
Other Race/Ethnicity (#/% students)	766 (10%)	722 (9%)
White (#/% students)	2,374 (30%)	2,444 (30%)
Female (#/% students)	3,860 (49%)	3,887 (48%)
Free-reduced lunch eligible (#/% students)	6,360 (80%)	6,308 (78%)
Special education eligible (#/% students)	1,219 (15%)	1,227 (15%)
English learner (#/% students)	1,516 (19%)	1,166 (14%)
Chronic health condition (#/% students)	1,614 (20%)	1,193 (15%)
**School level**		
Need index (M/SD)	0.50 (0.06)	0.48 (0.05)
Percent minority (M/SD)	0.71 (0.16)	0.69 (0.18)

Percentages may not sum to 100% due to rounding

Table 2 provides coefficients for our attendance and chronic absenteeism multilevel regression models and student- and school-level predictor variables (att.m2rs, chron.m2). ANOVA supported a random intercept, random slope (on prior year student-level attendance) attendance model over a random intercept model, χ^2^(2) = 139.09, *p* < 0.001, and the model with only student-level covariates, *χ*^*2*^(10) = 188.25, *p* < 0.001. The regression coefficient for *nurse* (*β* =  − 0.001, *S.E.* = 0.001, 95% CI [-0.003, 0.002], *p* = 0.41) indicated a statistically nonsignificant 0.10% decrease in attendance in schools with a nurse, suggesting that LPN presence was not associated with higher attendance as hypothesized. For chronic absenteeism, we preferred chron.m2 over a random intercept model with only student-level covariates, *χ*^*2*^(8) = 19.63, *p* = 0.01, and a random intercept, random slope model, *χ*^*2*^(2) = 1.02, *p* = 0.60. The statistically nonsignificant *nurse* coefficient (*β* = 0.12, *S.E.* = 0.07, 95% CI [− 0.02, 0.26], *p* = 0.10) indicated a 13% increase in the odds of being chronically absent. Thus, our hypothesized association between LPN presence and reduced chronic absenteeism was not supported. The model selection supplement (available online) provides R code and outputs for matching and model selection.

**Table 2:** Multilevel regression results for attendance and chronic absenteeism

Parameter	Model att.m2rs	Model chron.m2
**Fixed effects (*β*/*SE*)**^**a**^		
Intercept	0.95 (0.001)*	− 2.91 (0.10)*
17–18 attendance^b^	0.64 (0.01)*	
17–18 chron. abs		2.64 (0.04)*
Minority	0.01 (0.001)	− 0.39 (0.06)*
Male	0.000 (0.001)	− 0.05 (0.05)
FRL	− 0.005 (0.001)*	0.61 (0.08)*
Special Ed	− 0.003 (0.001)*	0.23 (0.07)*
EL	0.003 (0.001)*	− 0.36 (0.09)*
Health condition	− 0.004 (0.001)*	0.31 (0.06)*
Enrollment^b^	0.002 (0.001)	− 0.02 (0.04)
Percent reading novice^c^	− 0.01 (0.01)	1.08 (0.75)
Percent math novice^c^	− 0.003 (0.01)	− 0.07 (0.53)
Nurse	− 0.001 (0.001)	0.12 (0.07)
**Random effects (*SD*)**		
Intercept	0.003	0.13
17–18 attendance	0.08	
Residual	0.04	
**Fit statistics**		
*AIC*	60,637.91	9,936.85
*BIC*	60,484.27	10,067.45
Pseudo-*R*^*2*^	0.46	0.27

*β* regression coefficient, *SE* Standard Error, *SD* Standard Deviation, *AIC* Akaike Information Criterion, *BIC* Bayesian Information Criterion, *df* degrees of freedom

^a^Coefficients for balanced Level 2 variables used in matching are not reported

^b^Group mean centered

^c^Grand mean centered

^*^p < .05

### Costs

The district office incurred costs for an Assistant Superintendent’s time to oversee school health services; a district secretary and clerk to provide administrative support; six APRNs who provided supervision and training for school nurses and UAPs; three screening nurses who visited each school to conduct hearing, vision, and dental screenings; contract nurses who provided specific services for individual students as needed; and the Manager of District Health who monitored provision of health services, fulfilled reporting requirements, and supervised the APRNs, LPNs, and contract nurse services. Each school incurred costs for classified staff time for training and serving as UAPs. Schools with LPNs additionally bore the costs of the LPN while those without an LPN needed additional UAP time provided by certified staff. Adult family members contributed time to complete annual health forms and volunteers contributed time to conduct vision screenings. All schools received basic first aid supplies while schools with LPNs received a full suite of medical supplies (see Supply List tab in online supplemental Excel workbook). School classrooms were used for training activities. Schools with a nurse had fully equipped health offices while those without a nurse had health offices or sick rooms with only basic furniture and equipment. We did not have access to data on any external training activities in which nurses may have additionally engaged to maintain their professional licenses or on external health services to which students were referred by a school nurse.

We estimated that the nursing program in schools with LPNs cost, on average, $115,707 annually per school in 2018 dollars using national average prices. This serves as our reference case analysis (CASP, 2021) estimate of the school LPN program. Basic health services in comparison schools cost an average of $47,479 per school. Average incremental costs per school with an LPN were therefore $68,228. Table 3 shows the low, mean, and high costs per treatment and comparison school of itemized personnel and facilities ingredients as well as aggregated materials. The last column shows the incremental costs per ingredient of providing LPNs above and beyond the costs of basic health services. Costs per LPN school varied, primarily depending on the need for contract nurse services, the number of staff trained, the amount of volunteer time used for vision screening, and adult family member time used to complete health forms. Costs per LPN school ranged from $99,594 to $180,248, while costs per comparison school ranged from $33,211 to $87,677 (see Costs by sites T1 and Costs by sites C1 tabs in online supplemental Excel workbook). In Table 3, we indicate for each ingredient whether shifting one comparison school to the LPN program would result in an overall change in costs (i.e., is a variable cost (V)) or would remain unchanged (i.e., is a fixed cost (F)).

**Table 3:** Low, mean, high, and incremental costs per school (in US dollars) of ingredients needed to provide health services

Ingredient	National cost per school with LPN (treatment)				Total for T schools	National cost per school with no LPN (comparison)			Total for C schools	Incremental cost per school
	Low		Mean	High		Low	Mean	High		
Personnel: district office costs										
Asst. Supt., Academic Support (F)	67		109	148	2,517	59	109	215	2,517	
Manager of district health (F)	1,292		2,111	2,861	48,562	281	526	1,033	12,088	1,586
Clerk (F)	184		301	407	6,913	161	301	591	6,913	
Secretary (F)	185		303	410	6,964	162	303	595	6,964	
3 ES screening nurses (F)	873		1,427	1,934	32,829	764	1,427	2,805	32,829	
6 APRNs (F)	5,130		5,868	6,314	134,972	5,130	5,876	6,314	135,143	-7
Contract nurses (F)	630		8,826	75,826	203,006		6,460	45,830	148,584	2,366
Personnel: school costs										
Licensed practical nurses (V)	71,194		71,194	71,194	1,637,470					71,194
Certified UAPs (V)						8,982	8,982	8,982	206,587	-8,982
Classified UAPs (F)	5,650		5,650	5,650	129,955	5,650	5,650	5,650	129,955	-
UAP training (V)	203		1,234	2,530	28,375	289	1,211	2,625	27,863	22
Personnel: costs to others										
Volunteers for vision screening (F)	643		1,051	1,424	24,165	562	1,051	2,064	24,165	
Adult family members (F)	6,870		11,228	15,213	258,243	6,395	11,950	23,481	274,855	-722
Total personnel			109,303		2,513,971		43,846		1,008,462	65,457
Facilities: district office costs										
Office: Asst. Supt., AS (F)	1		1	2	25	1	1	2	25	
Office: MDH (F)	13		21	29	492	3	5	10	122	16
Office: clerk (F)	5		9	12	204	5	9	17	204	
Office: secretary (F)	5		8	11	184	4	8	16	184	
Facilities: school costs										
Sick room* (V)						1,481	1,481	1,481	8,886	-1,481
Health office (V)		2,527	2,527	2,527	58,110	2,527	2,527	2,527	42,951	
Training space (classroom) (V)		6	16	30	377	7	19	43	430	-2
Total facilities			2,582		59,393		2,296		52,803	287
M&E: district costs (mostly V)		1,174	1,210	1,242	27,830	87	295	377	6,792	915
M&E: schools costs (mostly V)		2,612	2,612	2,612	60,076	31	1,042	1,399	23,960	1,570
Total M&E		3,786	3,822	3,854	87,906	118	1,337	1,776	30,752	2,485
Grand total			115,707		2,661,270		47,479		1,092,017	68,228

*AS* Academic Services Division, *F* fixed cost that would not change if one comparison school shifts to the LPN program, *MDH* Manager of District Health, *M&E* materials and quipment, *V* variable cost that would change if one comparison school shifts to the LPN program

*Each school had either a sick room or a health office, but not both

Personnel time accounted for over 90% of the costs, with facilities and materials and equipment each accounting for 2–5%. The online supplemental Excel workbook shows additional cost breakdowns (see Summary tab Tables 1–11, Summary by resource category L(ocal) tab, Summary by resource category N(ational) tab, and Graphics tab). For example, Summary tab Table 11 shows costs by component and indicates that LPN services accounted for 66% of the nursing costs at the LPN schools. Our sensitivity analysis demonstrated that additional time spent by UAPs providing health services to students in the comparison schools resulted in a 19% increase in costs to these schools. Using local prices, we estimated average annual costs of $81,526 per LPN school and $38,947 per comparison school.

Combining results, we found mean per-school incremental costs of $68,228 produced no significant improvement in measured outcomes; thus, we do not report cost-effectiveness ratios. Based on 95% CIs for effects, our results suggest the incremental cost for LPNs was associated with, at best, 0.15% higher attendance (0.26 school days in a typical 175-day school year) and a 2.29% decrease in the odds of chronic absenteeism. At worst, LPN presence was associated with 0.34% lower attendance (0.60 school days) and a 29.69% increase in chronic absenteeism odds.

## Discussion

We conducted the first CEA of a school nursing program and the only known empirical investigation of an LPN-based school nursing program. In doing so, we answered calls for CEAs in nursing research (Bensink et al., 2013) and for methodologically rigorous school nursing studies investigating student educational outcomes (Best et al., 2018). Our study improves upon prior studies of RNs by investigating a large sample and using a more rigorous QED including a control group and controlling for confounding variables. Our costs of $115,707 per LPN school are substantially higher than prior published estimates of school nurse programs. After adjusting to 2018 dollars and converting local costs to a national equivalent using a regional price parity index from the Bureau of Economic Analysis, we equated Wang et al.’s (2014) estimate to $89,700 and Baisch et al.’s (2011) estimate to $96,900. These differences are not surprising, given Baisch et al.’s estimate focused solely on nurse salary and fringe benefits while Wang et al. added costs of medical supplies. Our results indicated that direct LPN costs accounted for only 66% of the total costs of the nursing program in LPN schools while supplies accounted for a mere 3%. Administration costs and basic health services constituted 10% and 14% of the total, respectively. We did not, however, investigate receipt of health services outside of school to assess whether students in comparison schools incurred greater external medical costs than students in LPN schools, which might have reduced our incremental cost metrics.

Our effectiveness analysis aimed to ascertain whether previously demonstrated positive effects of school-based RNs on student attendance and chronic absenteeism might also apply to school-based LPNs. Those questions seemed especially pertinent given the reliance on LPNs by schools in the southern USA prior to the COVID-19 pandemic (e.g., Willgerodt et al., 2018) and increased post-COVID hiring of school-based LPNs across the country (e.g., Buttner, 2021). Unfortunately, despite substantial investment in the JCPS school nurse program, we found at best negligible positive effects of LPNs on either outcome. One possible explanation is that school nursing alone may be insufficient to prevent student absences in a large, urban school setting. However, findings from Gottfried’s (2013) longitudinal study of Philadelphia elementary school nurses suggest this is not necessarily the case.

Our results may also be due to the limited training and scope of practice of LPNs relative to RNs (e.g., Buttner, 2021; Winsch, 2016), especially in leadership and care coordination (e.g., NASN, 2021). Our disappointing effectiveness findings complement previous positive RN results (e.g., Allen, 2003) in support of the NASN (2021) position that school nurses should be baccalaureate-trained RNs. Because JCPS did not have a monitoring plan in place for its LPN program, however, we were not able to obtain detailed information on LPN practices during the time of our study. As such, we examined the relationship between LPN *presence* (e.g., Darnell et al., 2019) and student academic outcomes. Therefore, we can only say with certainty that the LPN program *as implemented* was not associated with significantly higher attendance or reduced chronic absenteeism among elementary students, relative to using trained UAPs. We strongly caution against overinterpreting our results to suggest that school-based LPNs cannot be part of an effective strategy to improve attendance and against overgeneralizing our results to suggest limited LPN effects on alternative student health and academic outcomes.

To encourage further dialogue (e.g., Best et al., 2018), we can offer an alternative explanation for our findings that is centered on program implementation (i.e., ‘did not’) rather than intrinsic properties (i.e., “cannot”) of LPN-based nursing programs. The lack of standardized, district-specific school nurse training based on an explicit theory of change for educational outcomes and concomitant monitoring plan to ensure implementation fidelity may explain the observed ineffectiveness of the LPN-based program in this study. In other words, while JCPS acknowledged limitations of LPNs vs. RNs (Winsch, 2016), the district did not take steps to identify key LPN practices related to student attendance and chronic absenteeism and provide relevant training and monitoring, or to train and utilize its six APRNs wherever necessary to supplement LPN activities specifically aimed at improving those two outcomes (e.g., Buttner, 2021). The paucity of empirical guidance from school nurse researchers on best practices for improving attendance (e.g., Best et al.) hinders the development of logic models and training manuals for LPNs, although care coordination has at least some empirical support for improving attendance (e.g., Engelke et al., 2014).

Taken together, the above conditions likely resulted in considerable heterogeneity in the supervision and practice of the district’s LPNs. As such, the LPNs may have affected the health (and subsequent attendance) of only a subset of students not captured in our whole-school analysis. The LPNs may have also identified more student health conditions requiring treatment, the successful treatment of which may have increased absences even as student health improved. Finally, nurse presence in a school may increase absences through stricter adherence to immunization compliance and attendance policies (Winsch, 2016).

## Recommendations to Improve Cost-effectiveness of Nurse Programs

If schools and education agencies cannot provide a nurse in every school as proposed by the NASN (2021), we recommend developing a need-based process to assign nurses equitably to schools with students who could benefit most from this resource. School nursing programs may be less effective in preventing chronic absenteeism when students’ access to nursing services is inequitably distributed (e.g., Rankine et al., 2021), as was the case in our study. A careful review of the theory of change for nursing programs is needed to clarify what specific activities nurses should engage in and how their services are expected to lead to improved health outcomes that result in increased attendance, reduced absenteeism, and eventually improved student achievement. With a clearly specified theory of change, JCPS and other districts could provide initial training and continuing education (Rankine et al., 2021) based on a standard operating procedure manual that outlines the specific activities school nurses should conduct and the inputs required, plus suitable methods for monitoring and auditing. Finally, state and local education agencies in participating states can also shift some of the burden of school nursing costs to the federal government through expanded Medicaid billing eligibility (Buttner, 2021). Depending on an agency’s existing record-keeping and data infrastructure, additional upfront investment or consultation with an outside vendor may be needed to start the process.

## Limitations and Future Directions

Although we approximated random assignment with OMM, our algorithm may have failed to mitigate selection bias in nurse assignment. Because nurses were not assigned based on a needs assessment, our results may have been confounded by differences in relative nursing needs among treatment and comparison schools. We controlled for recorded student health conditions, but we did not directly observe student health and lacked granular receipt of health services data. If LPNs were assigned to schools with greater health needs, and if those needs were associated with poorer attendance, we could not rule out the possibility that LPNs were effective in stemming potentially worse attendance outcomes. On the other hand, some treatment school students may have experienced increased absenteeism because LPNs referred them to outside care. Our study may have also lacked sufficient statistical power to detect effects. Future research might address these issues by matching on student-level health data, measuring services received, and including more than 23 matched schools in MLM designs. Our quasi-experimental effectiveness study focused on the effects of LPN presence on elementary student attendance outcomes in one urban school district in the southeastern USA. Future studies should test the internal validity of our study design and MLMs using alternative student outcomes or employ experimental designs to determine the causal effects of specific school nursing activities and interventions on student health and educational outcomes across all grades and in different school contexts and locations. More research is also needed to understand the cost-effectiveness of LPNs vs. UAPs on health outcomes and LPNs vs. RNs on health and educational outcomes.

We assessed our CEA against the Standards for the Economic Evaluation of Educational and Social Programs (CASP, 2021) and found that it met most applicable standards. However, we conducted the cost analysis retrospectively rather than concurrently with implementation; that is, we collected data on the 2018–2019 implementation a year later (2019–2020). Given that the program is ongoing, and we had access to detailed school-level records maintained by the district on nursing services, we do not feel this was a major source of inaccuracy. That said, the range of UAP time spent on health services might have been better assessed through surveys or interviews. We also did not present results for different sub-groups of students because student-level data indicating which students received which health services were not available. We expect that students with health conditions would incur more costs for health services but might also have better attendance at schools with LPNs than similar students at schools with no nurse. A future study on the costs and benefits of school nurses could investigate these student-level effects and whether the provision of a school nurse leads to greater or less use of health services outside of school. These induced costs or savings would be, respectively, added to or subtracted from the school nursing costs. Finally, we assumed that nurses worked no volunteer hours. Including time logs in future studies could confirm this assumption or result in higher costs.

## Conclusion

We conclude that, counter to existing evidence in support of the positive effects of RN-based school nursing programs, the full-time LPN program at JCPS did not appear to be a cost-effective absence prevention strategy for improving elementary school student attendance and chronic absenteeism, despite substantial investment in the program. Our results suggest that LPN-based school nursing programs may need to provide additional training, monitoring, and auditing based on a well-developed theory of change to improve student attendance and chronic absenteeism. More research is needed to identify key nursing strategies and activities related to those outcomes and to understand whether improving existing LPN-based programs is cost-effective relative to implementing RN-based school nursing programs.

## Supplementary Information

Below is the link to the electronic supplementary material.

Supplementary file1 (DOCX 35 KB)

Supplementary file2 (XLSX 3044 KB)
