# Supplementary material for: Costs and Effects of School-Based Licensed Practical Nurses on Elementary Student Attendance and Chronic Absenteeism
Source: Prev Sci. 2022 Nov 17;24(1):94–104. doi: 10.1007/s11121-022-01459-0 (PMC9670058; doi:10.1007/s11121-022-01459-0)
Supplement: Supplementary file 1 — Supplementary file1 (DOCX 35 KB) [file 11121_2022_1459_MOESM1_ESM.docx]

**Costs and Effects of School-Based Licensed Practical Nurses on Elementary Student Attendance and Chronic Absenteeism: Matching and Model Selection Supplement^[[1]](#endnote-1)^**

**Matching**

Below is the R code used for optimal multilevel matching (OMM; Pimentel et al., 2018^[[2]](#endnote-2)^). The final set of *n* = 23 matched pairs reflects the largest set of schools with acceptable balance on prioritized school-level covariates: *need index, percent minority,* and *prior year attendance*. As seen in the output, matchMulti obtained acceptable balance (*asd* < 0.2) on those variables.

library(data.table)

library(matchMulti)

*#set student and covariates for matching*

stu.cov <- c("minority", "gender", "frl", "ECE", "LEP", "att1718")

sch.cov <- c("N_read", "N_math", "sch.minority", "need.index", "sch.att1718", "sch.chron1718")

*#nur.match is data set with treat schools & potential match schools, subset without kindergarten students*

nur.match.nok <- nur.match[grade!=0]

*#fine balanced matching using partitioned school-level covariates ######*

*#create nominal variables from continuous - using 2 achieved the best balance on the school level covariates*

nur.match.nok.hc$N_read_cut <- cut(nur.match.nok.hc$N_read, 2)

nur.match.nok.hc$N_math_cut <- cut(nur.match.nok.hc$N_math, 2)

nur.match.nok.hc$sch.minority_cut <- cut(nur.match.nok.hc$sch.minority, 2)

nur.match.nok.hc$need.index_cut <- cut(nur.match.nok.hc$need.index, 2)

nur.match.nok.hc$sch.att1718_cut <- cut(nur.match.nok.hc$sch.att1718, 2)

nur.match.nok.hc$sch.chron1718_cut <- cut(nur.match.nok.hc$sch.chron1718, 2)

*#match using student (no 1:1 on students) and school covariates (no kindergarten)*

*#preferred model matches on need.index_cut, sch.att1718_cut & sch.minority_cut first*

match.fb<-matchMulti(nur.match.nok.hc, treatment = 'nur.num', school.id = 'sln_mod', match.students = F,

verbose = T, student.vars = stu.cov,

school.fb = list(c('need.index_cut', 'sch.att1718_cut', 'sch.minority_cut'),

c('N_read_cut', 'N_math_cut', 'sch.minority_cut',

'need.index_cut', 'sch.att1718_cut', 'sch.chron1718_cut')), tol = .2)

*#check balance on school covariates (output is a table of asds)*

bal.tab.fb <- balanceMulti(match.fb,

student.cov = stu.cov,

school.cov = sch.cov)

out.fb <- cbind(bal.tab.fb$schools[,3],

bal.tab.fb$schools[,6])

colnames(out.fb) <- c("S.Diff Before", "S.Diff After")

round(out.fb, 3)

Table S1 below provides a comparison of the *asd*s of school-level covariates between treatment and comparison schools for our preferred matching versus preferred matching plus enrollment.

**Table S1**

| Covariate | Preferred Matching | |  | Preferred + enrollment |
| --- | --- | --- | --- | --- |
|  | *asd* before matching | *asd* after matching |  | *asd* after matching |
| *N_read* | 1.327 | 0.440 |  | 0.523 |
| *N_math* | 1.075 | 0.375 |  | 0.421 |
| ***sch.minority*** | 1.016 | 0.143 |  | 0.195 |
| ***need.index*** | 1.179 | 0.187 |  | 0.207 |
| ***sch.att.1718*** | -0.923 | 0.133 |  | -0.029 |
| *sch.chron1718* | 0.921 | -0.118 |  | 0.068 |
| *enroll* |  |  |  | -0.365 |

*Note*: *asd* = absolute standardized difference; covariates in bold were priority matching variables.

**Multilevel Regression**

To obtain ICCs for computing DE, we first estimated simple random intercept models with no covariates using the lme4 package. The R code for each model is provided below:

att.m0 <- lmer(att1819 ~ 1 + (1|sln_mod), data=nurse.mlm, REML = F) *#ICC = 0.0167*

chron.m0 <- glmer(factor(chron1819) ~ 1 + (1|sln_mod), data=nurse.mlm, family = binomial(logit)) *#ICC = 0.0198*

Because DEs supported using MLM (see manuscript for details), we next estimated random intercept models with only student-level covariates. The R code for each model is below:

att.m1 <- lmer(att1819 ~ 1 + att1718.c + minority + gender + frl + ECE + LEP + stu.hc +

(1|sln_mod), data=nurse.mlm, REML = F)

chron.m1 <- glmer(factor(chron1819) ~ 1 + factor(chron1718) + minority + gender + frl + ECE + LEP + stu.hc +

(1|sln_mod), data=nurse.mlm, family = binomial(logit))

We then compared model fit of att.m1 and chron.m1 with models that added school-level covariates. Per an anonymous reviewer, we included all six matching variables but only reported on unbalanced variables. Specifically, we estimated random intercept models (att.m2, chron.m2) with school-level covariates for enrollment (*enroll*; scaled per manuscript), *need index, percent minority,* *prior year attendance, prior year chronic absenteeism percentage,* *percent reading novice*, *percent math novice*, and *nurse*. The R code for each model is below:

att.m2 <- lmer(att1819 ~ 1 + att1718.c + minority + gender + frl + ECE + LEP + stu.hc +

scale(enrc) + N_readc + N_mathc + need.indexc + sch.minorityc + sch.att1718c + sch.chron1718c +

nurse + (1|sln_mod), data=nurse.mlm, REML = F)

chron.m2 <- glmer(factor(chron1819) ~ 1 + factor(chron1718) + minority + gender + frl + ECE + LEP + stu.hc +

scale(enrc) + N_readc + N_mathc + need.indexc + sch.minorityc + sch.att1718c +

sch.chron1718c + nurse + (1|sln_mod), data=nurse.mlm, family = binomial(logit))

Pairwise ANOVAs supported att.m2 vs. att.m1 (*χ^2^*(8) = 49.16, *p* < .001) and chron.m2 vs. chron.m1 (*χ^2^*(8) = 19.63, *p* = .01). We then estimated models based on the random intercept models above that also allowed prior year attendance (att.m2rs) and chronic absenteeism (chron.m2rs) slopes to vary randomly. The R code for those two models is below:

att.m2rs <- lmer(att1819 ~ 1 + att1718.c + minority + gender + frl + ECE + LEP + stu.hc +

scale(enrc) + N_readc + N_mathc + need.indexc + sch.minorityc + sch.att1718c +

sch.chron1718c + nurse + (1 + att1718.c|sln_mod), data=nurse.mlm, REML = F)

chron.m2rs <- glmer(factor(chron1819) ~ 1 + factor(chron1718) + minority + gender + frl + ECE + LEP + stu.hc +

scale(enrc) + N_readc + N_mathc + need.indexc + sch.minorityc + sch.att1718c +

sch.chron1718c + nurse + (1 + factor(chron1718)|sln_mod),

data=nurse.mlm, family = binomial(logit))

Pairwise ANOVA supported att.m2rs (same as Model 1a, but with *prior year attendance* slopes allowed to vary) over att.m2, *χ^2^*(2) = 139.09, *p* < .001 and over att.m1, *χ^2^*(10) = 188.25, *p* < .001. A similar chronic absenteeism model (chron.m2a) with random prior year chronic absenteeism slopes was not preferred over chron.m2, *χ^2^*(2) = 1.02, *p* = .60. Models att.m1, att.m2a, chron.m1, and chron.m2 are reported in the manuscript Results section and Table 3.

**Sensitivity Analyses**

Page, Lenard, and Keele (2020) recommend including in subsequent multilevel regression analyses only those unbalanced school-level variables used in the OMM algorithm (Pimentel, Page, & Keele, 2018). In our case, four school-level covariates were acceptably balanced and two were not; *percent reading novice* (*asd* = 0.44) and *percent math novice* (*asd* = 0.38), two variables indicating low proficiency on the state reading and math assessment, respectively. Therefore, we estimated we estimated versions (att.m2rs.sa, chron.m2.sa) of our preferred random intercept, random slope attendance model (att.m2rs) and random intercept chronic absenteeism model (chron.m2), but with each including only the school-level matching covariates that were unbalanced (plus all other non-matching covariates in the preferred models). The R code for the two sensitivity models is below:

model.2rs.sa <- lmer(att1819 ~ 1 + att1718.c + minority + gender + frl + ECE + LEP + stu.hc + scale(enrc) +

N_readc + N_mathc + nurse + (1 + att1718.c|sln_mod), data=nurse.mlm, REML = F)

chron.m2.sa <-glmer(factor(chron1819) ~ 1 + factor(chron1718) + minority + gender + frl + ECE + LEP + stu.hc +

scale(enrc) + N_readc + N_mathc + nurse + (1|sln_mod),

data=nurse.mlm, family = binomial(logit))

The coefficients of interest (i.e., *nurse*) with confidence intervals for these four models (att.m2a, att.m2a.s, chron.m2, chron.m2.s) are reported in Table S2 below with preferred models in bold.

**Table S2**

| Model | *nurse* (*β*/*SE*) | *p* | 95% CI |
| --- | --- | --- | --- |
| att.m2rs.sa | 0.0016 (0.0016) | .33 | [-0.0017, 0.0049] |
| **att.m2rs** | **-0.0010 (0.0012)** | **.41** | **[-0.0034, 0.0015]** |
| chron.m2.sa | 0.0630 (0.0746) | .40 | [-0.0832, 0.2092] |
| **chron.m2** | **0.1182 (0.0722)** | **.10** | **[-0.0232, 0.2597]** |

Using the 95% confidence intervals, the preferred attendance model (att.m2rs) predicts a slightly less desirable worst case (0.3% vs. 0.2% decrease in attendance, a difference of less than 1/3 day in a typical 175-day school year). The preferred attendance model also predicts a less desirable best case (0.2% vs. 0.5% increase in attendance, a difference of less than 2/3 day in a typical year). Similarly, the preferred chronic absenteeism sensitivity model predicted a slightly worse worst case (30% vs. 23% increase in the odds of being chronically absent, a difference of 7%) and a worse best case (2.3% vs. 8% decrease in the odds of being chronically absent, a difference 5.7%). Standard errors for preferred models are slightly smaller than for sensitivity models.

1. *frl* = free or reduced lunch-eligible; *ECE* = special education status; *LEP* = English Learner status; *stu.hc* = number of student health conditions. Variable descriptions are provided in the manuscript. [↑](#endnote-ref-1)
2. Pimentel, Page, & Keele (2018) provide a helpful R vignette complete with sample code and selected outputs at:

   https://cran.r-project.org/web/packages/matchMulti/vignettes/multiMatch_vignette.pdf [↑](#endnote-ref-2)
